# Contrasting mineralized and barren porphyries in the Zhongdian Arc, insights from biotite and apatite compositions and halogen fugacity

**DOI:** 10.1038/s41598-024-62120-0

**Published:** 2024-05-27

**Authors:** Yanning Pan, Guochen Dong, Toshiaki Tsunogae, Peng Wang, Xuefeng Li, Pengsheng Dong

**Affiliations:** 1 School of Earth Sciences and Resources, China University of Geosciences (Beijing), Beijing, 100083 China; 2 College of Geology and Environment, Xi’an University of Science and Technology, Xi’an, 710054 China; 3 School of Earth Science and Resources, Chang’an University, Xi’an, 710054 China; 4 Faculty of Life and Environmental Sciences (Earth Evolution Sciences), University of Tsukuba, Ibaraki, 305-8572 Japan; 5 Department of Geology, University of Johannesburg, Auckland Park, Johannesburg, 2006 South Africa; 6 School of Earth and Environmental Sciences, The University of Queensland, St Lucia, Brisbane, QLD 4072 Australia

**Keywords:** Pulang, Porphyry copper deposit, Biotite, Apatite, Halogen fugacity, Planetary science, Solid Earth sciences

## Abstract

Copper mineralization in the Pulang (PL) porphyry deposit, Langdu (LD) porphyry-skarn deposit and Songnuo (SN) porphyry prospect in northwestern Yunnan, China, is closely related to the emplacement of quartz monzonite porphyries. The chemical compositions of biotite and apatite from those porphyries were analyzed to calculate the halogen fugacity and to constrain mineralized and barren porphyries. Our data show that biotites from the PL deposit have higher MgO, SiO_2_, TiO_2_ and F contents than those from the LD deposit or SN prospect. Compared to those in the LD deposit and SN prospect, the Mg (atoms per formula unit (apfu)) and Al^VI^ (apfu) value in biotite is greater, and the F content is greater and the SO_3_ and Ce_2_O_3_/Y_2_O_3_ ratio in apatite are lower in the PL deposit. Ti-biotite thermometry and apatite-biotite geothermometry show that the crystallization temperature of biotite from the PL deposit is higher than that from the SN prospect or LD deposit. The results suggest that oxygen fugacity, magmatic sulfur, and H_2_O contents cannot be used to efficiently distinguish the PL deposit from the LD deposit and SN prospect. However, the halogen chemistry of biotite from the PL deposit is distinctly different from that of the LD deposit or SN prospect according to the lower IV (F), indicating that mineralized quartz monzonite porphyries in the PL deposit formed during the late magmatic stage, which is in contrast to those in the LD deposit and SN prospect. The mineralized porphyries display a remarkable negative linear relationship (r = − 0.96) with the log (*f* HF/*f* HCl) and log (*f* H_2_O/*f* HF) ratio, which can be used to distinguish the mineralized and barren porphyries. Compared with other typical porphyry Cu systems, there is a remarkable positive linear relationship between IV (Cl) and log (*f* H_2_O/*f* HCl). In addition, the linear slope and intercept for log (*f* H_2_O/*f* HCl) ratios and the IV (Cl) of biotite from the potassic and phyllic alteration zones are significantly greater than those from other porphyries.

## Introduction

Biotite, a common halogen-bearing ferromagnesian mineral in most porphyry deposits, forms during igneous crystallization of the host intrusive rock or during hydrothermal alteration^1–4^. The composition of magmatic biotite is sensitive to the chemical and physical factors associated with the crystallization of the magma and to the exsolved hydrothermal fluids^5^. Previous studies have suggested that the MgO, FeO, and Al_2_O_3_ contents of biotite are related to the original composition of the granitoid melt from which they crystallized^6,7^. Munoz^8,9^ showed that the FeO/(FeO + MgO) ratio of biotite is affected by the oxygen fugacity (*f*O_2_) of the system, which is a critical factor controlling the partitioning of Cu and Mo. In addition, theoretical estimates of F-Cl-OH partitioning between biotite and fluid (magmatic or hydrothermal)^10,11^, and new equations formulated to determine log (*f* HF/*f* HCl), log (*f* H_2_O/*f* HCl) and log (*f* H_2_O/*f* HF) fugacity ratios^9^ from biotite compositions, allow a more detailed evaluation of the chemistry of fluids(magmatic or hydrothermal) and their evolution within porphyry Cu systems. The halogen fugacity of magmatic and hydrothermal fluids, associated with mineralized porphyries and adjacent alteration zones in porphyry Cu (Mo, Au) deposits has been the focus of many studies^3,4,12–21^. It has also been demonstrated that the F and Cl contents in biotite from porphyry Cu deposits can be used to distinguish mineralized and barren plutons^22–24^. However, the potential of the halogen fugacity calculated by the F-Cl-OH of biotite as a porphyry deposit indicator has not yet been thoroughly evaluated.

Apatite is a common accessory mineral in igneous rocks and its F-Cl-S systematics can be used to investigate the F and Cl budgets of magmas and their source regions^25–27^. Apatite is also a major host for rare earth elements (REEs) and some large ion lithophile elements (LILEs), such as Sr, and as a result, apatite is a sensitive recorder of the trace element chemistry of magmatic systems^27–31^. Studies of apatite can be powerful for tracking key melt parameters, such as volatile contents^32–36^, which are important for assessing magmatic fertility. Consequently, apatite compositions have been proposed as another potential tool for identifying fertile porphyry copper intrusions from bare prospects.

The porphyry Cu deposit in Zhongdian is an integral part of the Tethys polymetallic belt^37,38^. A detailed investigation revealed the plutons, quartz monzonite porphyries, associated with the PL large scale porphyry Cu deposit^39–47^, LD medium porphyry skarn-type Cu deposit^48–51^ and SN porphyry Cu prospect^36,52,53^. However, the geochemical features of biotite and apatite from those mineralized and barren porphyries have not been studied. The relationship between the biotite fluorine—chlorine fugacity and mineralization in porphyry Cu deposits has also not been investigated. Hence, our work focus on the key question for a porphyry Cu deposit: how to distinguish the mineralized porphyry from the barren ones using biotite fluorine-chlorine fugacity. To answer this question, this paper presents new geochemical data on biotites and apatites from the PL and LD mineralized quartz monzonite porphyries, as well as from the SN barren quartz monzonite porphyry. By analyzing the compositions of biotite and apatite, we attempt to reveal (1) the halogen fugacity in the magmatic system associated with the PL and LD Cu deposits and the barren SN Cu prospect; (2) the crystallization temperature, pressure, sulfur-water content and *f*O_2_ of the magma; and (3) a key criterion for distinguishing the mineralized porphyry from barren porphyry.

## Geological setting

**Figure 1:**
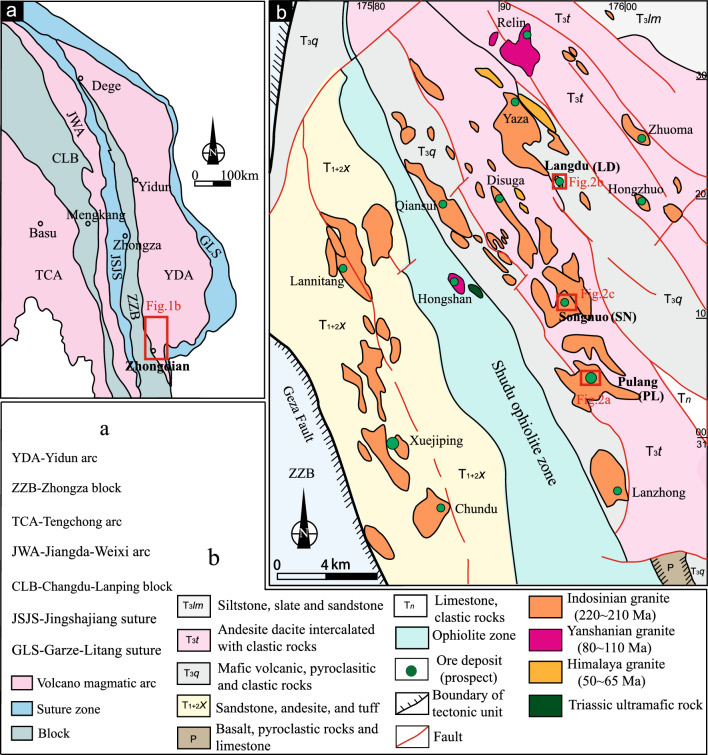
Tectonic setting of the Zhongdian island arc zone (**a** is modified from reference^57^) and regional geologic map (**b** is modified from reference^41^).

The Zhongdian porphyry copper deposit belt, located in Shangri-La County of northwest Yunnan Province, China^41^, is an integral part of the Sanjiang-Tethys polymetallic belt which is located in the eastern part of the Tethyan -Himalaya tectonic domain. The Zhongdian island arc is located at the southern end of the Yidun island arc^37,38^ (Fig. 1a). It is believed that the Garze-Litang Ocean subducted westward and formed the Yidun Arc on the eastern side of the Zhongza Block (Fig. 1a)^41,54^. The ages range from *ca.* 228–201 Ma, with a peak between 216 and 210 Ma^49,55^.

The tectonic evolution of the region witnessed two important stages. The Tethyan Ocean opened and closed during the Indosinian which induced subduction (or obduction) of the oceanic lithosphere^38,41,42^. A series of magmatic suites were generated through subduction- related processes, including dozens of Indosinian intrusions, some Yanshanian intrusions and a few Himalayan intrusions (Fig. 1b)^39,41,42,54,56^. The Indosinian intrusions in this area are typically associated with the PL super large porphyry copper deposit, LD medium-sized porphyry skarn-type copper deposit and SN porphyry copper prospect (Fig. 1b)^39,41,42,49^.

**Figure 2:**
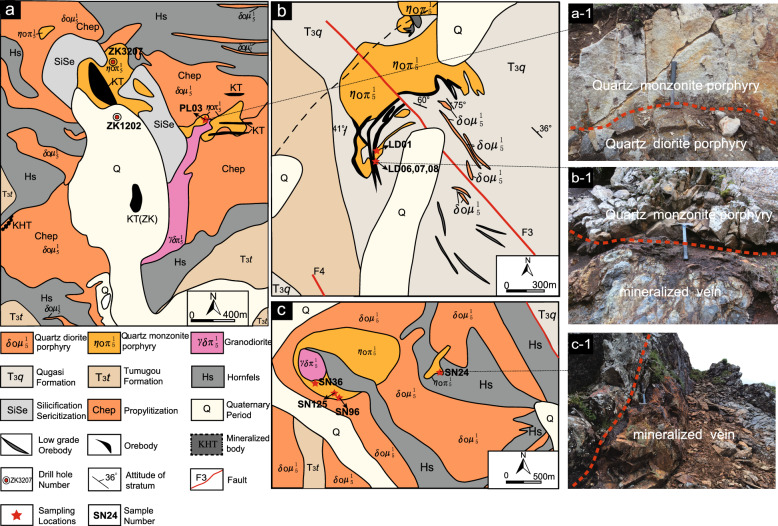
Geological maps of the studied deposits/prospect with sample locations and typical field photos (**a** is modified from reference^55^; **b** is modified from reference^48^).

The PL porphyry Cu deposit is related to a complex porphyry intrusion at 206 ~ 230 Ma^40–42,55,57–59^. The PL intrusion is composed of 5 main hypabyssal porphyries exposed over an area of 9 km^2^. These rocks were emplaced into the Tumugou Formation along NW-trending faults^55^ (Fig. 2a). Detailed field observations indicate that the PL complex can be divided into three distinct phases^57,58^ (Fig. 2a). The early phase is represented by quartz diorite porphyry, the middle phase by quartz monzonite porphyry and the late phase by granodiorite. Mineralization mainly occurs in quartz monzonite porphyry stocks^60^, with Cu reserves of more than 6.5 million tons, even in the PL deposit^58^.

The LD quartz monzonite porphyry, with an area of 0.9 km^2^, intruded into the Triassic Qugasi Formation and shows porphyry-skarn Cu mineralization (Fig. 2b) with Cu reserves of 10 thousand tons^48^. During the ore-forming process, the intrusive rocks were altered and skarns formed along the margins^49^. Our detailed field mapping revealed that the SN complex is composed of quartz diorite porphyry, quartz monzonite porphyry and minor granodiorite and has a lithology similar to that of the PL complex (Fig. 2c). Copper mineralization mainly occurs in the quartz monzonite porphyry^51^.

## Samples and analytical methods

### Samples

In total, 14 samples were collected in this study area during the field investigation. Three quartz monzonite porphyry samples in the PL deposit are from drill cores at different depths and one is from an exposure. Six samples of quartz monzonite porphyry in the SN prospect are associated with copper mineralization, and four samples of quartz monzonite porphyry in the LD deposit are from the intrusion margin, where some skarn is present. All the selected samples from both drill hole and outcrop were fresh or had weak alteration and were of a sufficient size. More than one kilogram of sample was collected from the outcrop. Detailed sample locations are provided in Fig. 2.

**Figure 3:**
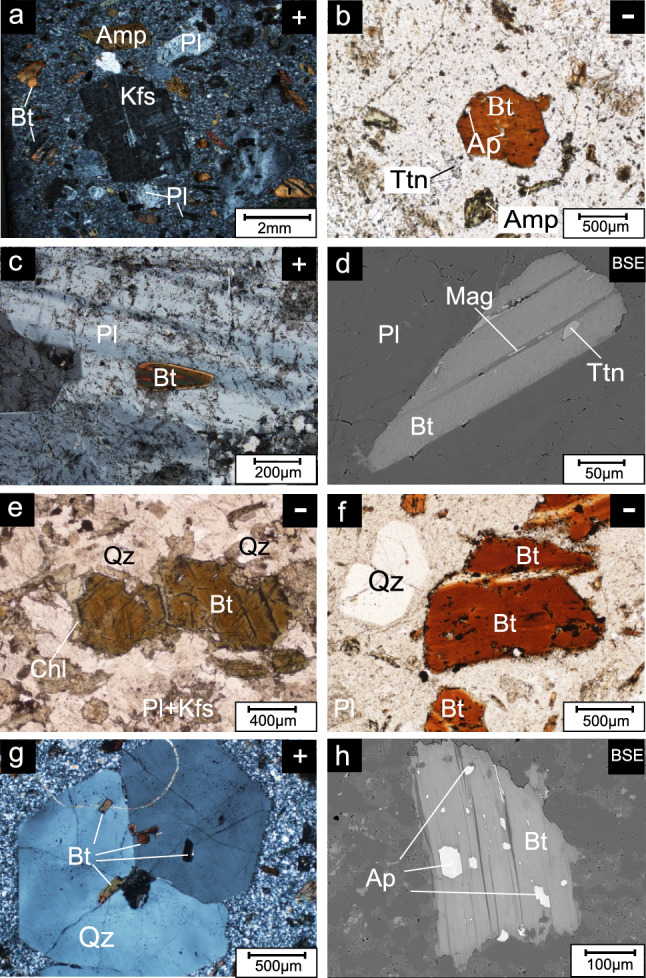
Petrographic characteristics of samples. (**a**) PL03 quartz monzonite porphyry with phenocrysts of plagioclase, K-feldspar, biotite and minor amphibole; (**b**) PL03 apatite coexisting with a biotite phenocryst; (**c**) PL3207 biotite inclusion in a plagioclase phenocryst; (**d**) PL3207 titanite and magnetite inclusion in biotite; (**e**) LD01 phenocryst biotite in a quartz monzonite porphyry of the LD deposit; (**f**) SN24 phenocryst biotite in a quartz monzonite porphyry of the SN prospect; (**g**) SN125 biotite inclusion in a quartz phenocryst; (**h**) PL1202 apatite occurring in the middle or margin of biotite; (−): plane polarized light; (+) cross polarized light; (BSE): back-scattered electron image; Pl: plagioclase; Bt: biotite; Amp: amphibole; Qz: quartz; Kfs: K-feldspar; Ap: apatite; Ttn: titanite. Mag: magnetite; Chl: chlorite.

The quartz monzonite porphyry in the PL deposit is gray in color and composed of phenocrysts of plagioclase (~ 5%), K-feldspar (~ 5%), biotite (5%), amphibole (0–3%) and quartz (0–2%) (Fig. 3a), which account for ~ 20% of the rock. And, the rock with minor accessory minerals of zircon, apatite and titanite. The biotite phenocrysts mainly form as 0.2–2 mm subhedral crystals and coexist with apatite (Fig. 3b). Some of them occur as inclusions in plagioclase (Fig. 3c,d).

The quartz monzonite porphyry in the LD deposit composed of phenocrysts of plagioclase (~ 5%), K-feldspar (~ 5%), amphibole (~ 5%), biotite (~ 3%) and quartz (~ 2%), which has more amphibole phenocrysts and fewer biotite phenocrysts and quartz. Most of the plagioclase phenocrysts are altered to sericite, whereas some biotite grains show marginal alteration to chlorite (Fig. 3e).

The quartz monzonite porphyry in the SN prospect is light-gray in color. The phenocrysts are dominated by quartz and biotite with little plagioclase (Fig. 3f). The quartz phenocrysts include microgranular inclusions of biotite (Fig. 3g). Apatite grains always occur in the middle or margin of the biotite (Fig. 3h).

### Analytical methods

Thin sections of the samples were cut for mineral identification under a microscope and microprobe. A total of 50g of powder (200mo) was selected for chemical analysis.

#### Major and trace element geochemistry

Quantitative analyses of major elements content in whole rocks performed by calibration curve method and conducted using X-ray fluorescence spectrometer (XRF), and, trace element concentrations were determined using inductively coupled plasma-mass spectrometry (ICP-MS) at the No.1 bureau of China metallurgical geology bureau, Hebei Province. For most of the trace elements, analytical precision and accuracy are better than 5%.

#### Electron microprobe analysis

To determine the key characteristics of the mineralized and barren intrusions, biotite, amphibole and apatite in quartz monzonite porphyry thin sections were selected for electron microprobe analysis (EMPA).

Biotite and amphibole were analyzed by using a JEOL JXA-8230 instrument. Analyses were undertaken by employing a 5 µm diameter electronic beam, for minerals with an accelerating voltage of 15 kV, a beam current of 20 nA and 10 s counting times for the peak and 5 s counting times for the upper and lower background per element. The microprobe analytical standards that were used were jadeite for Na and Al olivine for Mg, diopside for Si, and Ca, orthoclase for K, rutile for Ti, rhodonite for Mn, hematite for Fe, chromium oxide for Cr, nickel oxide for Ni, fluorite for F, and sodium chloride for Cl.

Chemical analyses of apatite were carried out by an electron microprobe analyzer (JEOL JXA-8621). Analyses were performed with a 3 µm diameter electronic beam under a 15 kV accelerating voltage and 10 nA sample current, and the data were regressed using an oxide- ZAF correction program supplied by JEOL.

## Results

### Whole-rock geochemistry

**Figure 4:**
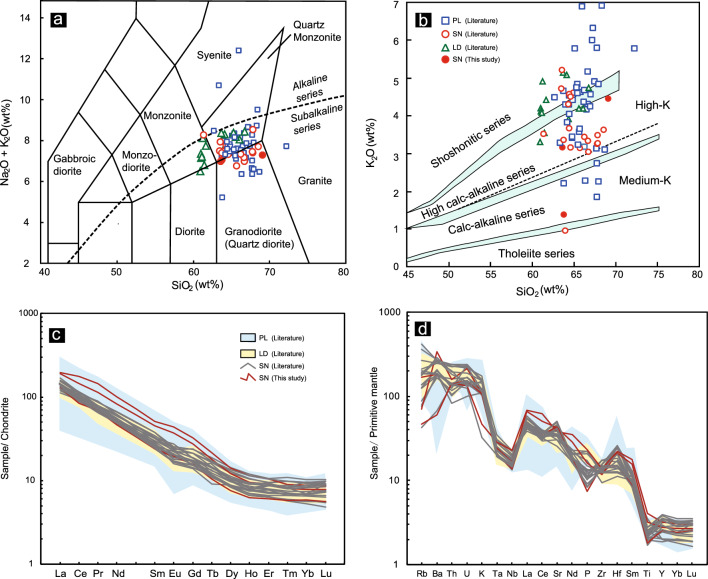
(**a**) (Na_2_O + K_2_O) versus SiO_2_ diagram for quartz monzonite porphyry (after reference^61,62^); (**b**) K_2_O versus SiO_2_ diagram (after reference^63^); (**c**) chondrite-normalized REE patterns; (**d**) primitive mantle-normalized multielement patterns. Normalization values for chondrite and primitive mantle are from reference^64^. The data were obtained from references^36,41,43,45,46,49–53,60^.

Whole-rock major and trace element data for 43 samples from the PL deposit, 14 from the LD deposit and 19 from the SN perspect, were compiled from the literature and the three new data for SN prospect are listed in Supplementary Table 1. The SN samples are composed of quartz monzonite and granodiorite and are the same as the PL porphyry, but the LD samples are composed of monzonite and quartz monzonite (Fig. 4a). The SN samples are of the high-K calc-alkaline to shoshonitic series (Fig. 4b), similar to porphyries from the PL and LD deposits. Porphyries from the SN prospect have high SiO_2_ (61.29–68.98 wt%, average = 65.23 wt%), Al_2_O_3_ (13.99–15.99 wt%, average = 14.96 wt%), and Sr (533–1052 ppm, average = 806 ppm) contents and La/Yb (19–34, average = 26) and Sr/Y (41–102, average = 64) ratios and low MgO (1.39–3.18 wt%, average = 2.09 wt%), Y (8.6–17.9 ppm, average = 13.2 ppm), and heavy REE (HREE) contents (Fig. 4c; Supplementary Table 1). These features are similar to those of the PL and LD deposits and other adakite-like porphyry intrusions in the Zhongdian Arc^41,43–45,49,53,59^.

In the chondrite-normalized REE diagrams, porphyries from the SN prospect, and PL and LD deposits display similar REE patterns (Fig. 4c). The SN porphyries are moderately enriched in light REEs (LREEs) and depleted in HREEs (La/Yb = 19–34), with slightly negligible Eu anomalies (0.76–1.09). These features are similar to those of porphyries from the PL and LD deposits. In the primitive mantle-normalized spider grams (Fig. 4d), the SN porphyries display obvious enrichments in large-ion lithophile elements (e.g., Rb, Th, U and K) and depletions in high field strength elements (e.g., Nb, Ta, and P). These features are also similar to those of the PL deposit and SN prospect. Consequently, whole-rock geochemistry is not efficient distinguishing the PL and LD deposits from the SN prospect.

### Mineral chemistry

#### Biotite

**Figure 5:**
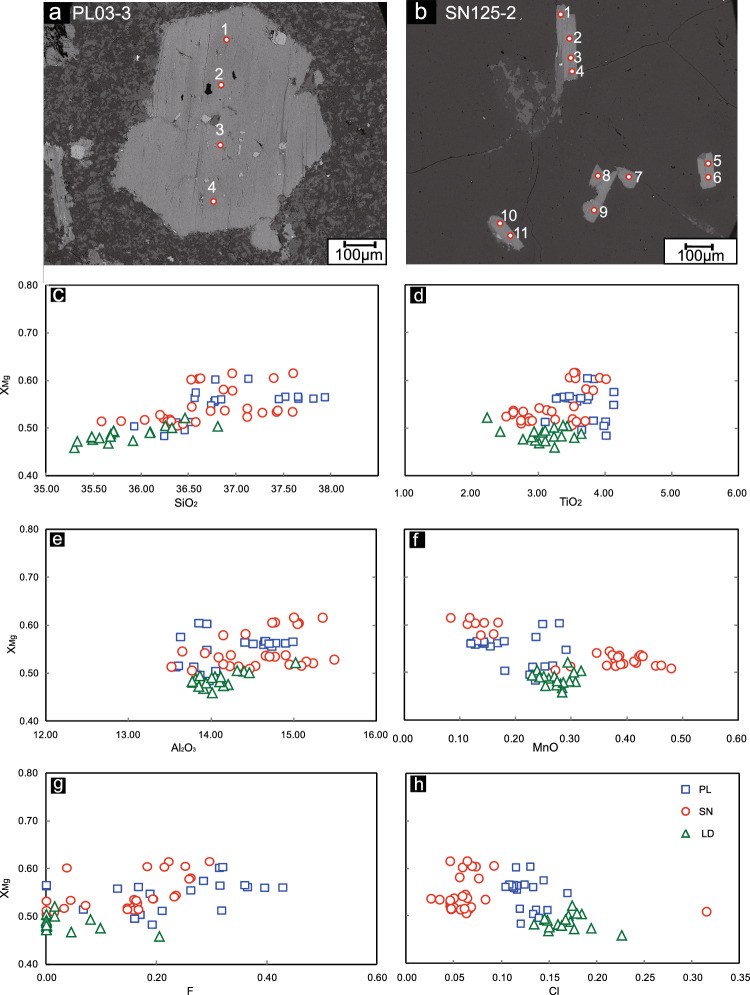
Results for the analyzed biotites from the PL and LD deposits and the SN prospect.

A total of 66 microprobe analyses of 11 biotite grains were performed on the porphyries from the PL deposit, LD deposit and SN prospect. The major element compositions of typical biotites from the PL deposit, LD deposit and SN prospect are shown in Table 1. The microprobe data are available in Supplementary Table 2. The representative biotite with tested points for electron probe method are shown in Fig. 5a,b.

**Table 1:** Electron microporbe analysis results of representative biotites in quartz monzonite porphyry from PL, LD deposits and SN prospect.

Sample	PL3207-3	PL3207-3	SN24-1	SN24-1	LD01-1	LD01-1
Rock type	QMP	QMP	QMP	QMP	QMP	QMP
Occurrence	Inclusion	Inclusion	Phenocryst	Phenocryst	Phenocryst	Phenocryst
No. of analyzed spots	1	2	1	2	3	4
SiO_2_	37.66	36.77	36.96	37.40	36.32	36.46
TiO_2_	3.44	3.57	3.56	3.46	3.23	2.23
Al_2_O_3_	14.89	14.73	15.34	14.76	14.45	15.01
FeO	16.49	16.61	14.61	15.14	19.15	18.69
MnO	0.17	0.15	0.08	0.17	0.30	0.29
MgO	12.01	11.76	13.21	13.21	10.97	11.63
Na_2_O	0.13	0.05	0.04	0.13	0.07	0.10
K_2_O	9.51	9.49	9.54	9.35	9.12	9.09
CaO	0.11	0.13	0.05	0.02	0.10	0.10
Cr_2_O_3_	0.10	0.12	0.06	0.05	0.01	0.02
NiO	0.01	0.04	0.00	0.00	0.02	0.05
F	0.37	0.26	0.22	0.25	0.02	0.02
Cl	0.11	0.12	0.05	0.09	0.17	0.17
Total	94.80	93.66	93.60	93.91	93.90	93.80
Based on O = 22						
Si	5.69	5.65	5.63	5.66	5.63	5.64
Al^IV^	2.31	2.35	2.37	2.34	2.37	2.36
Al^VI^	0.34	0.31	0.35	0.22	0.28	0.38
Ti	0.39	0.41	0.34	0.45	0.38	0.26
Fe^3+^	0.57	0.53	0.42	0.48	0.44	0.38
Fe^2+^	1.51	1.60	1.91	1.88	2.04	2.03
Mn	0.02	0.02	0.05	0.04	0.04	0.04
Mg	2.71	2.69	2.65	2.60	2.54	2.68
Ca	0.02	0.02	0.01	0.00	0.02	0.02
Na	0.04	0.02	0.02	0.03	0.02	0.03
K	1.83	1.86	1.83	1.82	1.81	1.79
Total	15.43	15.47	15.58	15.52	15.56	15.62
^T^Al	2.65	2.67	2.73	2.56	2.64	2.74
^T^Fe	2.08	2.13	2.34	2.36	2.48	2.42
F	0.18	0.13	0.11	0.12	0.01	0.01
Cl	0.03	0.03	0.01	0.02	0.04	0.05
OH	3.80	3.84	3.88	3.86	3.95	3.95

^1T^Al and ^T^Fe is the total number of Al and Fe atoms per amphibole unit formula.

^2^OH is calculated by OH = 4 − (Cl + F).

The biotites from the PL deposit show slightly higher MgO (average = 12.11%) than those from the LD deposit (average = 10.61%) or SN prospect (average = 11.73%). The biotite in the PL deposit also contains more SiO_2_ and TiO_2_ than those from the SN prospect and LD deposit (Fig. 5c,d). However, biotite from the SN prospect has an average Al_2_O_3_ content of 14.58%, which is much greater than that from the PL deposit (average of 14.25%) or LD deposit (average of 14.09%) and the MnO content has similar features (Fig. 5e,f). The FeO content of the biotite in the SN prospect (average of 17.00%) is lower than that from the PL deposit (average of 17.53%) and LD deposit (average of 19.61%). The F content of biotites from the PL deposit (average 0.23%) is typically greater than that from the SN prospect (average of 0.15%) or LD deposit (average of 0.03%) (Fig. 5g). However, biotites from the SN prospect typically contain less Cl than those from the PL or LD deposits (Fig. 5h).

In summary, the EPMA analyses show that biotites from the PL deposit have higher MgO, SiO_2_, TiO_2_ and F contents than those from the LD deposit or SN prospect, but the SN biotite has higher Al_2_O_3_ and MnO and lower FeO and Cl contents than those from the PL or LD deposit (Fig. 5).

**Figure 6:**
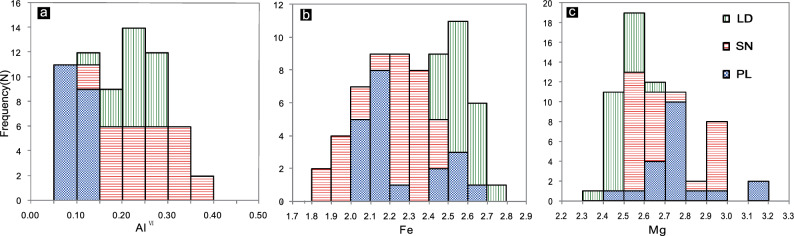
Histograms of electron microprobe biotite chemistry from the PL and LD deposits and SN prospect for (**a**) Al^VI^(apfu), (**b**) Fe (apfu), and (**c**) Mg (apfu).

The biotite grains from the PL deposit and SN prospect have X_Mg_ values of 0.48–0.60 and 0.51–0.62, respectively, which are higher than those from the LD deposit (0.46–0.59). Compared with those from the LD deposit (average of 0.25) and SN prospect (average of 0.31), the biotite from the PL deposit has a lower Al^VI^ content (average of 0.20) (Fig. 6a). The Fe (apfu) content of biotites is greater in the LD and PL deposits than in the SN prospect (Fig. 6b).

The PL biotite samples have Mg (apfu) values between 2.47 and 3.14, which are greater than those of the SN (2.52–2.99) and LD (2.36–2.68) samples (Fig. 6c). Thus, biotites from the PL deposit show higher Mg values and lower Al^VI^ values than those from the LD deposit or SN prospect (Fig. 6).

#### Apatite

A total of 53 microprobe analyses of 18 apatite grains were conducted on the porphyries from the PL deposit, LD deposit and SN prospect. The major element compositions of typical apatites from the PL deposit, LD deposit and SN prospect are shown in Table 2. The apatite microprobe data are given in Supplementary Table 3.

**Table 2:** Electron microporbe analysis results of representative apatites in quartz monzonite porphyry from PL, LD deposits and SN prospect.

sample	SN36	SN36	PL3207-2	PL3207-4	LD07	LD07	LD07	LD07
No. of samples	Ap-1 core 1	Ap-1 rim 3	32 Ap-1 core 1	32 Ap-1 rim 3	Ap-1 core 1	Ap-1 core 2	Ap-1 rim 3	Ap-1 rim 4
No. of analyzed spots	12	14	16	18	36	37	38	39
SiO_2_	0.37	0.34	0.49	0.34	0.24	0.41	0.35	0.42
MnO	0.13	0.09	0.07	0.17	0.02	0.06	0.11	0.12
CaO	53.95	53.78	54.36	54.66	54.38	54.21	53.66	53.80
Ce_2_O_3_	0.08	0.20	0.26	0.27	0.16	0.50	0.43	0.45
SO_3_	0.55	0.42	0.72	0.20	0.22	0.47	0.36	0.31
BaO	0.10	0.04	0.04	0.10	0.11	0.07	0.04	0.14
Y_2_O_3_	0.17	0.14	0.16	0.21	0.24	0.27	0.31	0.27
P_2_O_5_	41.68	41.15	41.03	41.07	41.54	41.04	40.56	40.90
SrO	0.06	0.08	0.07	0.06	0.00	0.00	0.00	0.03
F	3.82	4.11	3.63	3.49	3.32	3.06	2.78	2.87
Cl	0.04	0.08	0.02	0.19	0.10	0.12	0.68	0.63
F/Cl	87	52	191	18	34	26	4	5
Based on O = 12.5								
Si	0.03	0.03	0.04	0.03	0.02	0.03	0.03	0.04
S	0.03	0.03	0.05	0.01	0.01	0.03	0.02	0.02
Y	0.01	0.01	0.01	0.01	0.01	0.01	0.01	0.01
Ce	0.00	0.01	0.01	0.01	0.00	0.02	0.01	0.01
Mn	0.01	0.01	0.00	0.01	0.00	0.00	0.01	0.01
Sr	0.00	0.00	0.00	0.00	0.00	0.00	0.00	0.00
Ba	0.00	0.00	0.00	0.00	0.00	0.00	0.00	0.00
Ca	4.87	4.91	4.92	4.98	4.94	4.93	4.94	4.92
P	2.97	2.97	2.93	2.95	2.98	2.95	2.95	2.96
Total	7.93	7.96	7.96	8.01	7.97	7.97	7.98	7.98
F	1.02	1.11	0.97	0.94	0.89	0.82	0.76	0.78
Cl	0.01	0.01	0.00	0.03	0.01	0.02	0.10	0.09
OH	0.98	0.88	1.03	1.03	1.10	1.16	1.14	1.13
S in melt	0.025	0.011	0.073	0.003	0.003	0.015	0.007	0.005
S in melt	0.048	0.036	0.077	0.021	0.014	0.029	0.022	0.019

**Figure 7:**
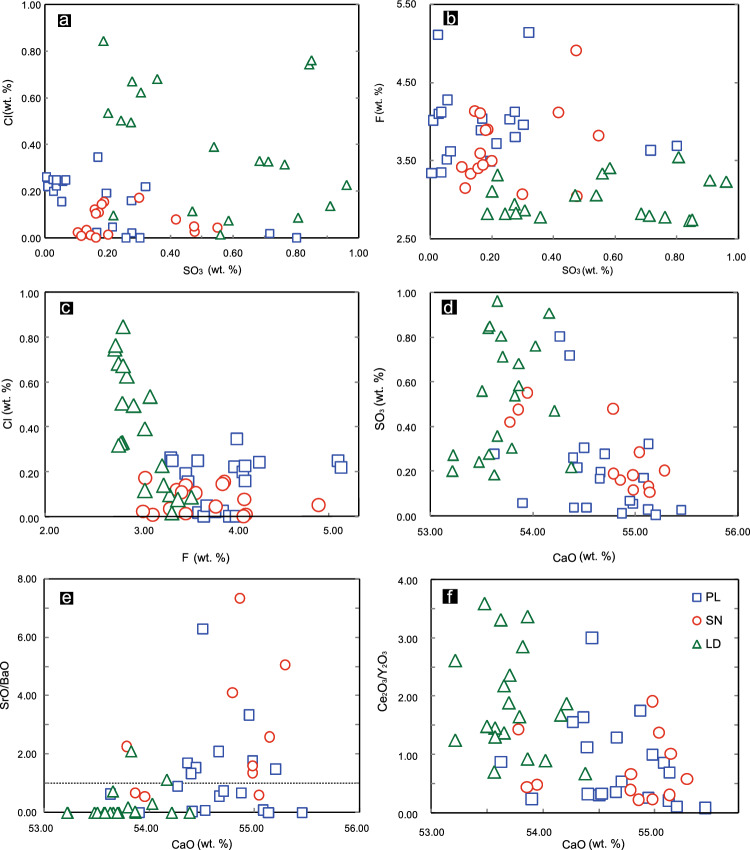
Results of the analysis of apatites from the PL and LD deposits and from the SN prospect.

The results show that the PL and LD deposits have hydroxyl apatite, and some fluorapatite, but the SN prospect samples all have hydroxyl apatite^65^. The apatites from the PL deposit have lower SO_3_ contents than those from the LD deposit or SN prospect (Fig. 7a,b).

However, apatites from the PL deposit show an average F content of 3.95%, which is higher than that from the SN prospect (average = 3.62%) and LD deposit (average = 3.02%) (Fig. 7b). The average apatite Cl content of 0.08% from the SN prospect is lower than that from the PL (average of 0.15%) and LD deposit (average of 0.40%) (Fig. 7a). Similar to SO_3_ and CaO content, the F and Cl contents of apatite from the three localities show a negative relationship (Fig. 7c,d).

Apatite is an important carrier of U, Th, Sr and REEs and is a sensitive recorder of trace elements in magmatic systems^27,66^. Apatites from the PL and LD deposits have higher Ce_2_O_3_ and Y_2_O_3_ contents than those from the SN prospect, but the SN apatite has a higher SrO content than that from the PL or LD deposits. The data show a positive correlation between the CaO content and the SrO/BaO ratio (Fig. 7e). The average SrO/BaO ratio of 2.62 in apatite from the SN prospect is significantly higher than that from PL (average of 1.16) or LD (< 1.0). In contrast, the data show a negative correlation between CaO and Ce_2_O_3_/Y_2_O_3_ values (Fig. 7f). The average Ce_2_O_3_/Y_2_O_3_ ratios of the PL deposit, SN prospect and LD deposit are 0.82, 0.87 and 1.87, respectively.

#### Amphibole

**Figure 8:**
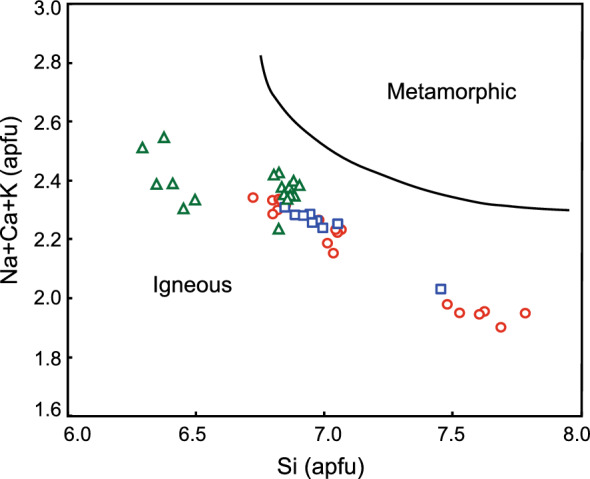
Na + K + Ca versus Si diagram after reference^67^; the symbols are the same as in Fig. 7.

A total of 46 microprobe analyses of 10 amphibole grains were obtained from the porphyries from the PL deposit, LD deposit and SN prospect. The chemical analyses of amphiboles are listed in Supplementary Table 4. The amphiboles from the PL deposit, LD deposit and SN prospect samples plot in the field of igneous rocks on the Na + K + Ca versus Si diagram^67^ (Fig. 8). The amphiboles from the PL deposit, LD deposit and SN prospect by Hawthorne^68^ and Oberti^69^, calculated by the Locock^70^ spreadsheet, are ^W^ (OH, F, Cl) group and Ca-amphibole subgroup. The species are mainly magnesio-ferri-amphibole and some actinolite. The results show that the amphibole from SN show relatively higher SiO_2_ contents than those from LD or PL. SN amphibole also contain much MgO than that from PL or LD.

### Physicochemical conditions

#### Temperature and pressure

Three independent geothermometers were used: a Ti-in-biotite thermometer, an amphibole thermobarometer and an apatite-biotite geothermometer.

(1) Ti-in-biotite thermometry

The Ti concentration in biotite is very sensitive to temperature and oxygen fugacity. Therefore, biotite can be used as a geothermometer in igneous and metamorphic rocks^71^. The Ti-in-biotite (T_Ti-in-biotite_) thermometer was calculated according to Henry^71^, and the geothermometry was formulated by the following expression:

1 $${\text{T}} = {\left\{ {\left[ {{\text{ln}}\left( {{\text{Ti}}} \right)-{\text{a}}-{\text{c}}{{\left( {{{\text{X}}_{{\text{Mg}}}}} \right)}^3}} \right] \, /{\text{b}}} \right\}^{0.333}}$$

in which T is the temperature in degrees Celsius, Ti is the number of atoms per formula unit (apfu) normalized on the basis of 22 O atoms, X_Mg_ is Mg/(Mg + Fe), a = − 2.3594, b = 4.6482 × 10^–9^ and c = − 1.7283. The calibration range for this expression is X_Mg_ = 0.275–1.000, Ti = 0.04–0.60 apfu, and T = 480–800 °C. The standard deviation of the Ti‐in‐biotite geothermometer is estimated to be ± 24 °C at temperatures of 480–600 °C ± 23 °C at 600–700 °C and ± 12 °C at 700–800 °C^71^.

The T_Ti-in-biotite_ yields ranged from 693 to 743 °C (average = 722 °C), 662 to 746 °C (average = 703 °C) and 642 to 713 °C (average = 688 °C) for the PL deposit, SN prospect and LD deposit, respectively (Supplementary Table 2). The Ti-in-biotite thermometry data suggest that the magma for the PL biotites in porphyries formed at higher temperatures than that for the SN and LD biotites.

(2) Amphibole thermobarometer

The amphibole thermometer of Ridolf^72^ was used to estimate the T of the magma from which amphiboles crystallize, where:

2 $${\text{T}} = - 151.487{\text{Si}}* + 2041 \, \left( {{\delta_{{\text{est}}}} = \pm \, 22 \, ^\circ {\text{C}}} \right)$$

For the detailed error (δ_est_) and expressions of Si* see Ridolf^72^. The results for the amphibole thermometer yielded values ranging from 715 to 812 °C (average of 788 °C), 650 to 839 °C (average of 771 °C) and 808 to 938 °C (average of 855 °C) for the PL deposit, SN prospect and LD deposit, respectively (Supplementary Table 4). Thus, the data suggest that the magma for the PL and LD amphiboles crystallized at higher temperatures than that for the SN amphiboles.

**Figure 9:**
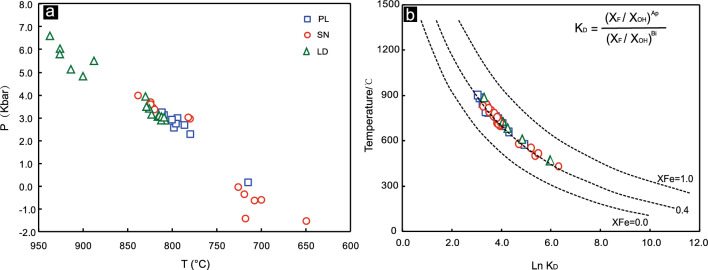
(**a**) Crystallization pressure (*P*) versus temperature (*T*) diagram after reference^10,11^ of amphiboles from the PL and LD deposits and SN prospect. (**b**) Equilibrium temperature calculated by an apatite-biotite geothermometer for the PL and LD deposits and SN prospect.

The pressure of amphibole crystallization was estimated using the Al-in-amphibole geo-barometer^73^:

3 $${\text{P }}\left( {{\text{kbar}}} \right) \, = \, - \, 3.01 \, + \, \left( {4.76 \, \times {\text{ A}}{{\text{l}}^{{\text{total}}}}} \right) \, \left( {{\delta_{{\text{est}}}} = \, \pm \, 0.6\;{\text{kbar}}} \right)$$

where Al^total^ is the total number of Al atoms per amphibole unit. The calculated pressures from the PL deposit, LD deposit and SN prospect are 2.3 to 3.2 kbar at depths of 9–12 km, 2.7 to 6.6 kbar at depths of 10–25 km and 2.6 to 4.0 kbar at depths of 10–15 km, respectively. The average pressures for the crystallization of amphiboles from the PL deposit, LD deposit and SN prospect are 2.8, 4.0 and 3.2 kbar, respectively (Supplementary Table 4). The amphibole thermobarometer results show that the temperatures and pressures for crystallization of amphiboles from the LD deposit is greater than that from the PL deposit or SN prospect, which is different from Ti-biotite thermometry results. In our investigation, amphibole only occurred as penocryst in the quartz monzonite porphyry form the PL and LD deposit and SN prospect. Hence, we infer that magmatic differentiation for ore-forming materials was proceeded after amphibole crystallized. In addition, there is a clear trend of increasing temperatures and decreasing crystallization pressures from the LD and PL deposits to the SN prospect (Fig. 9a).

(3) Apatite-biotite geothermometer

The apatite-biotite geothermometer by Stormer and Carmichael^74^ is based on the partition coefficient of F-OH between apatite and biotite. A revised apatite-biotite geothermometer was formulated by the following equation^10,11^:

4 $${\text{T }}{(^\circ }{\text{C}}) = 8852 - 0.024{\text{P}}\left( {{\text{bars}}} \right) + 5000{{\text{X}}_{{\text{Fe}}}}/\left( {1.987\;{\text{Ln}}\;{{\text{K}}^{\text{Ap/Bt}}}_{\text{D,F}} + 3.3666} \right) - 273.15$$

in which X_Fe_ = (Fe + Al^VI^) / (Fe + Mg + Al^VI^), Ln K^Ap/Bt^_D,F_ (Fig. 9b). This equation is only good for the T range of 300–1100 °C^10,11^.

The pressure mentioned in the equation above is calculated by Al-in-hornblende barometry (Eq. (3), Schmidt^73^). The results show average pressures of 2.8 kbar, 3.2 kbar and 4.0 kbar for the PL deposit, SN prospect and LD deposit, respectively. Thus, the average equilibrium temperatures are estimated to be 787 ℃, 742 ℃ and 662 ℃ for the PL deposit, SN prospect and LD deposit, respectively. Consequently, the data suggest that the magma for the crystallization of biotite and apatite from the PL deposit formed at higher temperatures than that from the SN prospect and LD deposit (Fig. 9b).

#### Oxygen fugacity and H_2_O contents of magma

**Figure 10:**
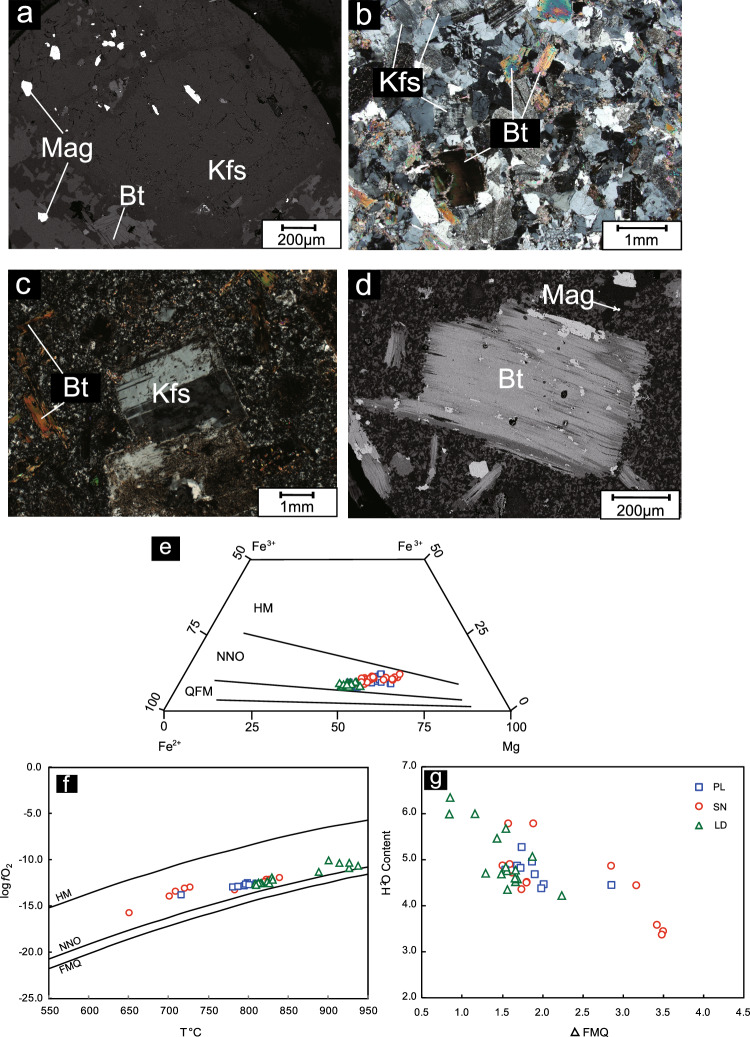
(**a**–**d**) Cogenetic assemblage of biotite-magnetite-K-feldspar under microsxope (**a** PL1202; **b** LD07; **c**,**d** SN36); (**e**) Fe^3+^–Fe^2+^–Mg diagram (after reference^75^), QFM = quartz-fayalite- magnetite buffer, NNO = nickel-oxide nickel buffer, and HM = hematite-magnetite buffer; (**f**) Log *f* O_2_ versus temperature diagram (after reference^44^). Temperature is calculated by Eq. (2), and log* f*O_2_ is calculated by the method proposed by Ridolfi^72^; (**g**) H_2_O content vs. ΔFMQ diagram. The H_2_O content is calculated by Eq. (6). The ΔFMQ is calculated by Geo-* f* O_2_ software developed by Li^76^.

Biotites from the PL deposit, LD deposit and SN prospect, which formed under the conditions of a cogenetic biotite–magnetite–K-feldspar assemblage (Fig. 10a–d), can be used to calculate the oxygen fugacity as proposed by Wones and Eugster^75^. The compositions of biotites from the PL deposit, LD deposit and SN prospect plot between those of the QFM and HM buffers in the Fe^3+^–Fe^2+^–Mg diagram of Wones and Eugster^75^, which implies a high oxygen fugacity (Fig. 10e).

Ridolfi^72^ produced the ΔNNO–Mg* oxybarometer, ^VI^Al*‐sensitive hygrometer to estimate the *f* O_2_, and H_2_O contents of magma, respectively, from which amphibole crystallizes, as described by the following formulae:

5 $$\Delta {\text{NNO}} = \left( {1.644 \times {\text{Mg}}*} \right) - 4.01 \, \left( {\delta {\text{est }} = \pm \, 0.22{\text{ log unit}}} \right)$$

6 $${{\text{H}}_{2}}{{\text{O}}_{{\text{melt}}}} = \left( {5.215 \, { \times^{{\text{VI}}}}{\text{Al}}*} \right) + 12.28 \, \left( {{\delta_{{\text{est}}}} = \pm \, 0.4 \, \;{\text{wt}}\% } \right)$$

The error (δ_est_) and Mg* and ^VI^Al* can be estimated by the method of Ridolf^72^ and ΔNNO can be calculated and converted into ΔFMQ by the method of Li^76^. The logƒO_2_ contents for amphibole crystallization in the PL deposit, LD deposit and SN prospect range from − 13.7 to − 12.4, − 12.7 to − 10.0 and − 15.7 to − 11.9, respectively, and the oxygen fugacity of the parental magma for the PL deposit, LD deposit and SN prospect is estimated based on the log ƒO_2_-T diagram^76^ (Fig. 10f). The ΔFMQ values in the PL deposit, LD deposit and SN prospect range from 1.5 to 2.8, 0.8 to 2.2 and 1.5 to 3.5, respectively (Supplementary Table 4). The H_2_O contents calculated by Eq. (6) for amphibole crystallization from the PL deposit, LD deposit and SN prospect samples range from 4.4 to 5.3, 4.2 to 6.4 and 3.4 to 5.8, respectively. As a result, the data suggest that ΔFMQ and magmatic H_2_O contents cannot efficiently be used to distinguish the PL deposit from the LD deposit and SN prospect. However, there is a negative correlation between the oxidation state and magmatic water content based on the LD, PL and SN data (Fig. 10g).

#### Sulfur fugacity

The sulfur partition coefficient between apatite and melt is jointly controlled by temperature, oxygen fugacity, and the S content in silicate melt^34,77,78^, and no formula exists to calculate magmatic sulfur concentrations from apatite SO_3_ contents^34^. However, two formulae can be used to estimate the relative magmatic S content^77,78^. Parat^78^ calibrated the following empirical non-Henrian partitioning relationship for sulfur between apatite and melt:

7 $${{\text{S}}_{{\text{apatite}}}}\left( {{\text{wt }}\% } \right) = 0.0629 \times {\text{ ln }}{{\text{S}}_{{\text{melt}}}}\left( {{\text{wt}}\% } \right) + 0.4513$$

The results calculated by the method of Parat^78^ show that the magmas in the PL and LD deposits have average sulfur concentrations of 0.012 and 0.071, respectively, which are higher than those in the SN prospect (average of 0.007) (Supplementary Table 3).

The temperature-dependent apatite–melt partition coefficient formula by Peng is used^77^:

8 $${\text{Ln}}\;{K_{\text{d}}} = 21130/{\text{T}}-16.2$$

where T is in Kelvin, and the temperature is calculated by an apatite-biotite geother- mometer^10,11^, The results show average temperatures of 787 ℃, 742 ℃ and 662 ℃ for the PL deposit, SN prospect and LD deposit, respectively. Thus, the magmas related to the PL deposit, LD deposit and SN prospect are calculated to have sulfur concentrations of 0.022, 0.034 and 0.024, respectively. The magmatic sulfur contents were estimated in absolute values by two methods^32–36^. These results suggest that the magmatic sulfur content could not efficiently distinguish the PL and LD deposits from the SN prospect.

#### Halogen fugacity

In muscovite- and fluorite-free granitoid rocks, 70 to 90% of the F is hosted in biotite, and the others are hosted in apatite and titanite^4,21,79^. Because the ionic radius of Cl^−^ (1.81 Å) is larger than that of F (1.31 Å) or OH (1.38 Å)^8^, the amount of Cl substitution in the OH site is less than that in F. The extent of halogen replacement of the hydroxyl group in biotite is governed by its Mg/Fe ratio. To calculate the relative degree of halogen enrichment in biotite, intercept values (IV), IV (F), IV (Cl), and IV (F/Cl) were developed by Munoz^8^ to correct for the effect of Mg/Fe ratios via the following equations:

9 $${\text{IV}}\;\left( {\text{F}} \right) = 1.52 \, {{\text{X}}_{{\text{phl}}}} + 0.42 \, {{\text{X}}_{{\text{ann}}}} + 0.20 \, {{\text{X}}_{{\text{sid}}}} - {\text{ log }}\left( {{{\text{X}}_{\text{F}}}{/}{{\text{X}}_{{\text{OH}}}}} \right)$$

10 $${\text{IV }}\left( {{\text{Cl}}} \right) = - 5.01 - 1.93{{\text{X}}_{{\text{phl}}}} - {\text{log }}\left( {{{\text{X}}_{{\text{Cl}}}}{/}{{\text{X}}_{{\text{OH}}}}} \right)$$

11 $${\text{IV }}\left( {\text{F/Cl}} \right) = {\text{IV }}\left( {\text{F}} \right) - {\text{IV }}\left( {{\text{Cl}}} \right)$$

where X_phl_ = Mg/sum of octahedral cations; X_sid_ = [(3 − Si/Al)/1.75] (1 − X_phl_); and X_ann_ = 1 − (X_phl_ + X_sid_). These equations show that IV(F) and IV(Cl) have negative correlations with X_F_ and X_Cl_^8^. The intercept value represents high halogen enrichment in the biotites.

**Figure 11:**
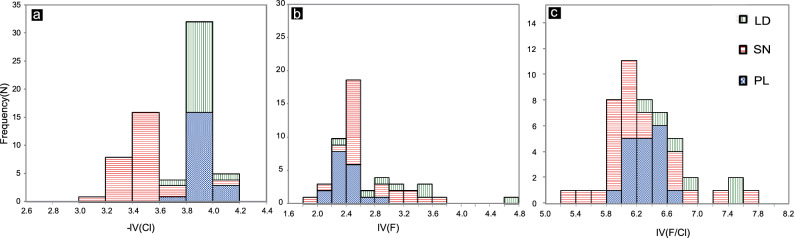
Histograms of (**a**) chlorine [-IV(Cl)], (**b**) fluorine [IV(F)], and (**c**) F/Cl intercept values [IV (F/Cl)] for biotites from the PL and LD deposits and SN prospect.

The biotite in the PL deposit is distinct from that in the SN prospect and LD deposit because of its low IV (F), with an average of 2.43 (Fig. 11). The biotites have average IV (F) values of 2.71 for the SN prospect, and 3.27 for the LD deposit. However, the SN prospect has an average IV (Cl) of -3.50 and a lower IV (F/Cl) of 6.21 than does the PL and LD deposits (Fig. 11). The IVs (Cl) and IVs (F/Cl) of the biotites in the PL deposit are on average − 3.88 and 6.32, respectively, and the IVs (Cl) and IVs (F/Cl) of the biotites in the LD deposit are on average − 3.90 and 7.18, respectively.

The fluorine and chlorine contents in biotite can be used to calculate the halogen fugacity in associated magma or fluids^8,9,12,13,16–18,21^. The following equations were used by Munoz^9^ based on the revised coefficients for F-Cl-OH partitioning between biotite and hydrothermal fluid^10,11^:

12 $${\text{Log }}{\left( {f{{\text{H}}_{2}}{\text{O}}/f{\text{HF}}} \right)^{{\text{fluid}}}} = 1000/{\text{T }}\left( {2.37 + 1.1 \, {{\text{X}}_{{\text{phl}}}}} \right) + 0.43 - {\text{log }}{\left( {{{\text{X}}_{\text{F}}}{/}{{\text{X}}_{{\text{OH}}}}} \right)^{{\text{biotite}}}}$$

13 $${\text{Log }}{\left( {f{{\text{H}}_{2}}{\text{O}}/f{\text{HCl}}} \right)^{{\text{fluid}}}} = 1000/{\text{T }}\left( {1.15 - 0.55 \, {{\text{X}}_{{\text{phl}}}}} \right) + 0.68 - {\text{log }}{\left( {{{\text{X}}_{{\text{Cl}}}}{/}{{\text{X}}_{{\text{OH}}}}} \right)^{{\text{biotite}}}}$$

14 $${\text{Log }}{\left( {f{\text{HF/}}f{\text{HCl}}} \right)^{{\text{fluid}}}} = - 1000{\text{/T }}\left( {1.22 + 1.65 \, {{\text{X}}_{{\text{phl}}}}} \right) + 0.25 + {\text{log }}{\left( {{{\text{X}}_{\text{F}}}{/}{{\text{X}}_{{\text{Cl}}}}} \right)^{{\text{biotite}}}}$$

where X_F_, X_Cl_, and X_OH_ are the mole fractions of F, Cl, and OH in the hydroxyl site of biotite, respectively, and X_phl_ = Mg/sum of octahedral cations. In this study, the T for the equilibrium temperature is an average of 722 ℃ in the PL deposit, 703 ℃ in the SN prospect and 688 ℃ in the LD deposit via the Ti-in-biotite geothermometer^71^.

The calculated log (*f* H_2_O/*f* HF), log (*f* H_2_O/*f* HCl), and log (*f* HF/*f* HCl) ratios of the magmatic fluids are shown in Supplementary Table 2. The average log (*f* H_2_O/*f* HF) ratio is 4.88 in the PL deposit, which is lower than that in the SN prospect (5.22) or LD deposit (5.83). The average log (*f* H_2_O/*f* HCl) ratio of 3.76 in the SN prospect is greater than that in the PL (3.64) or LD (3.57) deposits. The value of log (*f* HF/*f* HCl) is an average of -1.15 in the SN prospect, which is higher than that in the LD (-1.37) or PL (-1.24) deposits.

## Discussions

### Petrogenetic implications

**Figure 12:**
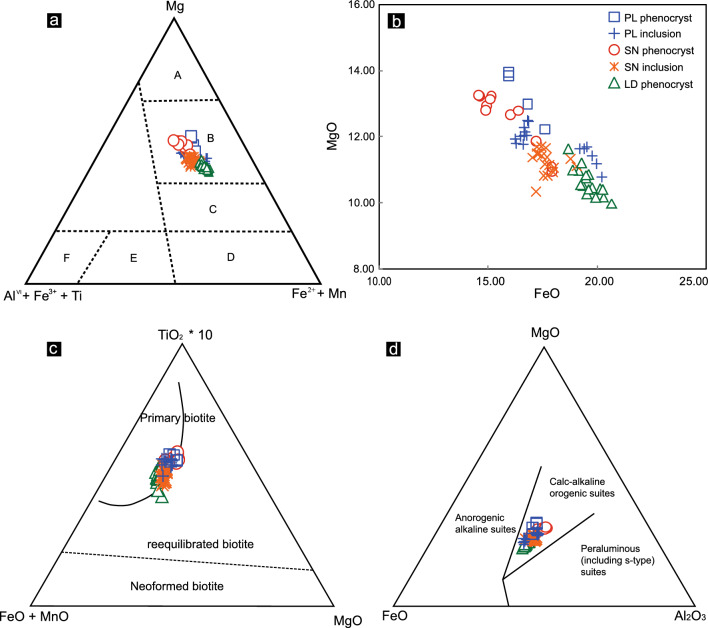
(**a**) Classification of phenocryst and inclusion biotites from the PL and LD deposits and SN prospect based on chemical and lithological affinities (adapted from reference^83^). A Phlogopites; B Mg-biotites; C Fe-biotites; D siderophyllites, lepidomelanes; E Fe-muscovites; F Muscovites. (**b**) Comparison diagram of the MgO-FeO of biotites. (**c**) Ternary TiO_2_-FeO + MnO-MgO diagram after reference^82^; (**d**) Ternary MgO-FeO-Al_2_O_3_ diagram after reference^6^.

Biotites from the PL deposit and SN prospect occur as phenocrysts and inclusions. In the Mg–(Al^VI^ + Fe^3+^  + Ti)–(Fe^2+^  + Mn) ternary diagram for the classification of trioctahedral micas by chemical and lithological affinity, all the biotites plot in the “Mg-biotites” field, which includes Mg dominant trioctahedral micas (Fig. 12a). However, the phenocryst biotites from the PL deposit and SN prospect show higher MgO and lower FeO contents than the biotites in inclusions (Fig. 12b). It is worth mentioning that biotite chemical composition may reflect magmatic conditions of crystallization. The analyzed biotites are of primary magmatic origin^21^. The Ti content of biotite is thermally controlled. Primary magmatic biotites have more Ti than re-equilibrated and neo-formed biotites^80,81^. Most of the phenocryst and inclusion biotites from the three intrusions plot in the primary biotite domain defined by Nachit^82^ (Fig. 12c), with Ti contents (apfu) ranging from 0.36 to 0.48 (average = 0.42) for the PL deposit, from 0.29 to 0.46 (average = 0.38) for the SN prospect, and from 0.26 to 0.43 (average = 0.36) for the LD deposit.

In a study by Abdel-Rahman^6^ on the composition of biotite from various igneous rock types, it was shown that biotites in alkaline anorogenic suites are mostly iron-rich, those in peraluminous suites are siderophyllitic in composition, and those in calc-alkaline, mostly subduction-related orogenic suites are enriched in Mg. The MgO-FeO-Al_2_O_3_ diagram is shown in Fig. 12d. All biotite grains from the three intrusions plot within the domain of calc-alkaline orogenic suites. This interpretation is supported by their whole-rock geochemistry, with high SiO_2_, Al_2_O_3_ and Sr contents and La/Yb and Sr/Y ratios and low MgO, Y and heavy REE (HREE) contents (Supplementary Table 1), showing adakitic affinities and indicating a subduction setting.

**Figure 13:**
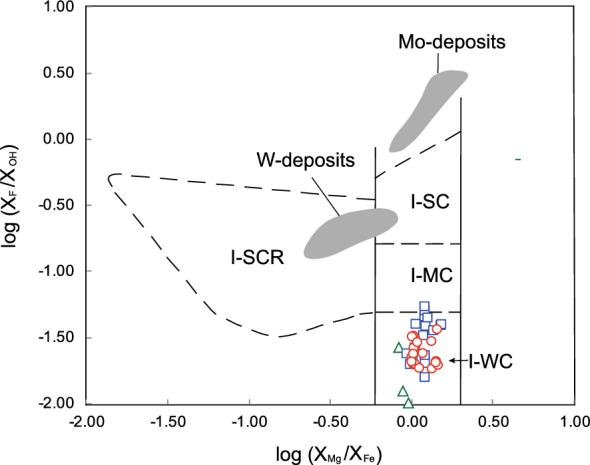
Classification of the quartz monzonite porphyry from the PL and LD deposits and SN prospect according to the compositions of their biotites (after reference^85^). For comparison purposes, the biotites related to the Mo- and W-porphyry deposits are also shown after reference^86^. I-SC, strongly contaminated I-type; I-MC, moderately contaminated I-type; I-WC, weakly contaminated I-type; I-SCR, strongly contaminated and reduced I-type. See Fig. 10 for the symbols used.

Ague and Brimhall^84,85^ studied the relationship between Mg/Fe and F/OH in magmatic biotite and related these ratios to the degree of contamination of the igneous rocks with continental crust. The rocks containing biotite with log (X_Mg_/X_Fe_) < − 0.21 are classified as strongly contaminated and reduced I-type, whereas the rocks containing biotite with log (X_Mg_/X_Fe_) >  − 0.21 are classified as oxidized and divided into three subgroups based upon increasing F/OH: (1) weakly contaminated I-type (I-WC); (2) moderately contaminated I-type (I-MC); and (3) strongly contaminated I-type (I-SC). The three subgroups of I-type granitoids are characterized by a high oxidation state^86^. The plot of log (X_Mg_/X_Fe_) and log (X_F_/X_OH_) of the biotites indicates that the quartz monzonite porphyries from the PL deposit, LD deposit and SN prospect are categorized as close to oxidized I-WC (Fig. 13). This interpretation is supported by whole- rock geochemistry, Sr–Nd isotopes and zircon ε_Hf_(t) and δ^18^O values from previous studies, indicating that porphyries in the PL deposit, LD deposit and SN prospect are I-type granites derived from mantle and crustal sources^43,44,46,50,52^.

### Metallogenic implications

#### Implication of oxygen fugacity

Most porphyry copper deposits (PCDs) are genetically associated with relatively highly oxidized magmas^87^. A high oxygen fugacity (ƒO_2_) is one of the important causal factors controlling the formation of PCDs and epithermal deposits because the migration and speciation of sulfur and subsequent sulfide stability in magma are directly affected by oxygen fugacity. In other words, sulfur dissolves in silicate melts in the form of sulfate (SO_4_^2-^ ) and sulfur dioxide (SO_2_) under high oxygen-fugacity conditions, which are favorable for copper transport as chlorine complexes^3,88,89^.

The biotite compositions in the PL deposit, LD deposit and SN prospect plot above those in the NNO buffers in log ƒO_2_ versus temperature and Fe^3+^–Fe^2+^–Mg diagrams (Fig. 10e, f), suggesting that the porphyry in the PL deposit, LD deposit and SN prospect formed at high oxygen fugacities^75^. Therefore, the hydrothermal fluids derived from this magma with relatively high oxygen fugacity could have elevated copper contents in the porphyries.

In addition, the biotites from productive granitic porphyry generally have higher Fe^3+^/Fe^2+^ and X_Mg_ ratios than those from barren granitic porphyry^3^. The Fe^3+^/Fe^2+^ ratios of the biotites ranges from 0.18 to 0.38, with an average of 0.28 in the PL deposit, from 0.16 to 0.22 (average of 0.19) in the LD deposit, and from 0.22 to 0.45 with an average of 0.29 in the SN prospect. The X_Mg_ values of the biotites are between 0.48 and 0.60, with an average of 0.55 in the PL deposit and 0.46 and 0.52, with an average of 0.49 in the LD deposit, while the X_Mg_ values of the biotites in the SN prospect are between 0.51 and 0.62, with an average of 0.55. It is suggested that the Fe^3+^/Fe^2+^ ratio and X_Mg_ value of biotite may not be able to be used to effectively distinguish the PL deposit from the LD deposit and SN prospect.

#### Implication of halogen fugacity

**Figure 14:**
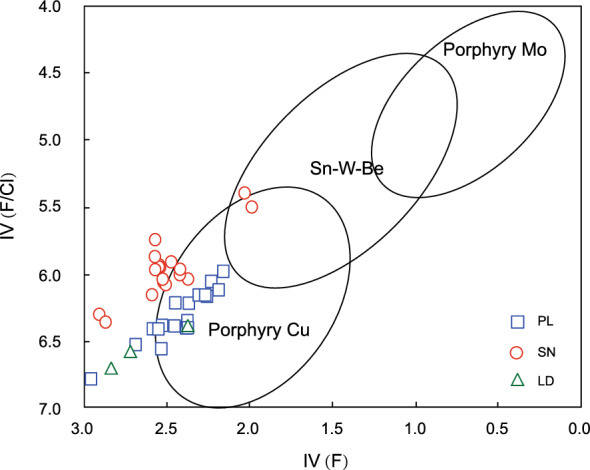
Intercept value IV(F/Cl) against IV(F) plot for biotites from the PL deposit and SN prospect and LD deposit. The composition of biotites related to typical porphyry Cu, Sn-W–Be, and porphyry Mo deposits are from reference^8^.

The F/Cl intercept values for biotites from typical porphyry copper deposits, Mo-W-Sn-Be deposits, and the Henderson porphyry molybdenum deposit plotted against IV (F) after Munoz^8^ are used here for comparison. In the IV (F/Cl) versus IV (F) diagram (Fig. 14), biotite in the PL deposit shows more F enrichment (low IV (F)), and most of the data plot in the porphyry copper area, which is distinct from the biotites in the SN prospect and LD deposit. The decrease in the IV(F) value might be caused by crystal fractionation, during which F is concentrated in the late magmatic stage^12^. Thus, the porphyries in the PL deposit associated with copper mineralization formed in a later magmatic stage than those in the LD deposit and SN prospect in the Zhongdian PCD belt.

**Figure 15:**
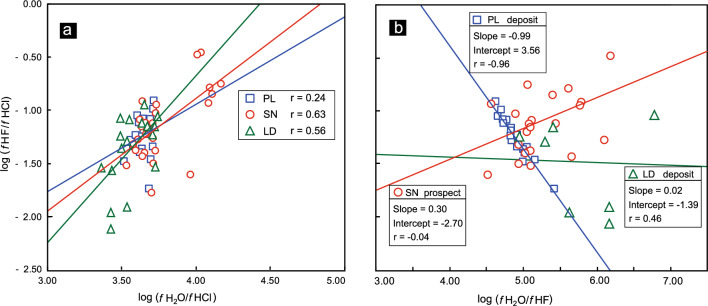
(**a**) Log (*f* HF/*f* HCl) vs. log (*f* H_2_O/*f* HCl) and (**b**) log (*f* HF/*f* HCl) vs. log (*f* H_2_O/*f* HF) for biotites from the PL and LD deposits and SN prospect. All the calculated formulae for log (*f* H_2_O/*f* HCl), log (*f* H_2_O/*f* HF), and log (*f* HF/*f* HCl) values are from references^8,9^.

Alternatively, the halogen ratios of biotites may not be able to effectively distinguish mineralized systems from barren systems^8^. However, in this study, the log (*f* H_2_O/*f* HF), log (*f* H_2_O/*f* HCl), and log (*f* HF/*f* HCl) ratios of magmatic fluids in equilibrium with magmas, which are derived from F–Cl–OH partitioning between biotite and the fluid, provide key criteria for distinguishing mineralized rocks from barren intrusions. The results show that the linear correlation coefficients between the log (*f* HF/*f* HCl) and log (*f* H_2_O/*f* HCl) ratios of the PL deposit, SN prospect and LD deposit are 0.24, 0.63 and 0.56, respectively (Fig. 15a). In contrast, the log (*f* HF/*f* HCl) and log (*f* H_2_O/*f* HF) ratios from the PL deposit show remarkable negative linear correlations, which are significantly different from those from the LD deposit and SN prospect (Fig. 15b). The linear correlation coefficients between log (*f* HF/*f* HCl) and log (*f* H_2_O/*f* HF) ratios of the LD deposit and SN prospect are − 0.04 and 0.46, respectively, which are significantly lower than the value of − 0.96 for the PL deposit (Fig. 15b). Thus, in the Zhongdian PCD belt, we note a remarkable negative linear relationship between log (*f* HF/*f* HCl) and log (*f* H_2_O/*f* HF) ratios from the mineralized PL intrusions, in sharp contrast with the barren SN intrusions.

#### Comparison of fugacity ratios with those of other porphyry Cu systems

**Figure 16:**
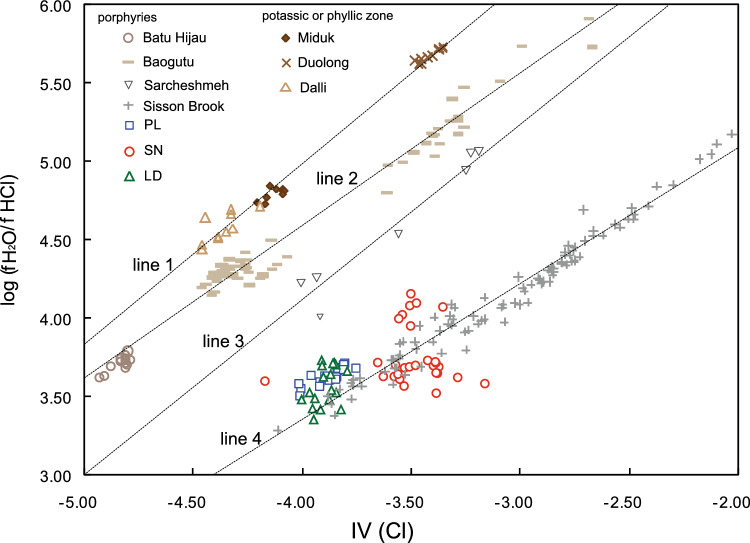
Comparison of the PL and LD deposits and SN prospect with other porphyry systems in terms of IV (Cl) vs. log (*f* H_2_O/*f* HCl) fugacity ratios.

The chlorine intercept [IV (Cl)] is an important physicochemical parameter for describing the relative degree of Cl enrichment in biotite, which is related to the fugacity ratio, *f*HCl/ *f*H_2_O. The log (*f* H_2_O/*f* HCl) ratios were strongly positively and linearly correlated with IV(Cl) (Fig. 16), with linear correlation coefficients between IV(Cl) and log (*f* H_2_O/*f* HCl) of 0.95, 0.97, 0.81, 0.67, 0.80, 0.99 and 0.98 for Duolong^90^, Sarcheshmeh^18^, Batu Hijau^15^, Miduk^17^, Dalli^16^, Baogutu^91^ and Sisson Brook^21^ deposits, respectively.

In Zhongdian, the IV(Cl) and log (*f* H_2_O/*f* HCl) ratios from the PL PCD also have a remarkable positive linear correlation, with a correlation coefficient of 0.86. The IV(Cl) is also positively and linearly correlated with the log (*f*_H2O_/*f*_HCl_) ratio for the LD PCD to a moderate degree (r = 0.42). However, the IV(Cl) and log (*f*_H2O_/*f*_HCl_) ratios for the SN prospect do not show a linear correlation (Fig. 16). Thus, it is apparent that a remarkable positive relationship between the IV(Cl) and log (*f*_H2O_/*f*_HCl_) ratios can be used to effectively distinguish mineralized systems from barren systems.

**Figure 17:**
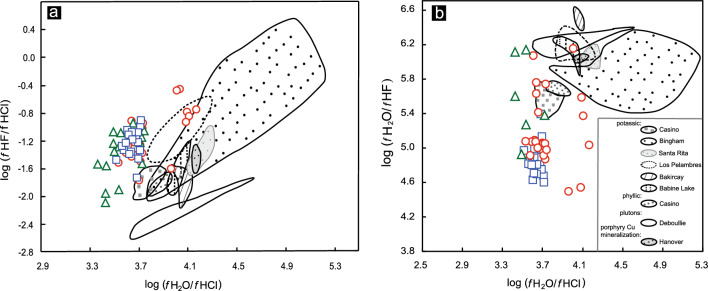
Comparison of the PL and LD deposits and SN prospect with those of other porphyry systems in terms of: (**a**) log (*f* HF/*f* HCl) vs. log (*f* H_2_O/*f* HCl), and (**b**) log (*f* H_2_O/*f* HF) vs. log (*f* H_2_O/*f* HCl) fugacity ratios (after reference^13^).

The calculated halogen fugacities from biotites for the Duolong, Dalli and Miduk deposits were from potassic or phyllic alteration zones, while those for the Sarcheshmeh, Batu Hijau, Baogutu, Sisson Brook and PL deposits were all from porphyries closely related to copper mineralization. The linear slope and intercept between the log (*f*_H2O_/*f*_HCl_) ratios and IV(Cl) for the Duolong, Dalli and Miduk deposits (line 1) are 1.16 and 9.61, respectively, which are greater than those of the Batu Hijau and Baogutu deposits (line 2: slope = 0.98, intercept = 8.49), Sarcheshmeh (line 3: slope = 1.11, intercept = 8.58) and Sisson Brook (line 4: slope = 0.88, intercept = 6.82) deposits. Therefore, the results show that the linear slope and intercept for log (*f*_H2O_/*f*_HCl_) ratios and IV(Cl) from the potassic and phyllic alteration zones are significantly greater than those from the porphyries (Fig. 16). Selby and Nesbitt^13^ compared the calculated fugacity ratios of hydrothermal fluids associated with potassic and phyllic alteration and porphyry plutons and reported that log (*f*_H2O_/*f*_HCl_) ratios from potassic and phyllic alteration zones are significantly greater than those from porphyries (Fig. 17). The variation shown in the log (*f*_H2O_/*f*_HCl_) values among the PCDs may be directly related to the composition of the exsolved magmatic aqueous fluids, which may in turn depend on magma processes (assimilation/fractional crystallization) during their ascent through the crust^13^.

## Conclusions

1. Biotites from the PL deposit with high MgO, SiO_2_, TiO_2_ and F contents display higher Mg (apfu) values and lower Al^VI^ (apfu) values than those from the LD deposit or SN prospect. The apatite in the PL deposit is distinct from that in the LD deposit or SN prospect because of its high F but lower SO_3_ and Ce_2_O_3_/ Y_2_O_3_ ratios.
2. Biotite Ti thermometry and apatite-biotite geothermometry show that the crystallization temperature from the PL deposit is greater than that from the SN prospect or LD deposit. The Al-in-amphibole geobarometer shows that the pressure in the PL deposit is lower than that in the LD deposit or SN prospect, indicating that the quartz monzonite porphyry from the PL deposit formed in the lower paleodepth than that from the LD deposit or SN prospect.
3. The halogen chemistry of biotite in the PL deposit is distinctly different from that in the LD deposit or SN prospect because of its lower IV(F), which means that the mineralized porphyries in the PL deposit formed in a later magmatic stage than those in the LD deposit and SN prospect.
4. The mineralized porphyry dikes display a remarkable negative linear relationship (r = − 0.96) with the log (*f* HF/*f* HCl) and log (*f* H_2_O/*f* HF) ratios, which possibly indicates intensive mineralization. Halogen fugacity can be used to distinguish the PL deposit from the LD deposit and SN prospect effectively via this linear pattern.

## Supplementary Information

Supplementary Information 1.

Supplementary Information 2.

Supplementary Information 3.

Supplementary Information 4.

## Data Availability

All data generated or analysed during this study are included in this published article and its supplementary information files.
